# *Coverage and Cost-of-Care* Links: Addressing Financial Toxicity Among Patients With Hematologic Cancer and Their Caregivers

**DOI:** 10.1200/OP.22.00665

**Published:** 2023-03-08

**Authors:** Jean S. Edward, Laurie E. McLouth, Mary Kay Rayens, Lori P. Eisele, Tani S. Davis, Gerhard Hildebrandt

**Affiliations:** ^1^ College of Nursing, University of Kentucky, Lexington, KY; ^2^ Department of Behavioral Health, College of Medicine, University of Kentucky, Lexington, KY; ^3^ Patient Financial Experience, University of Kentucky HealthCare, Lexington, KY; ^4^ Markey Cancer Center, Division of Hematology and Blood and Marrow Transplants, Lexington, KY; ^5^ Ellis Fischel Cancer Center, Missouri University Health Care, Columbia, MO

## Abstract

**PURPOSE:**

This study examined the feasibility, acceptability, and preliminary effectiveness of an oncology financial navigation (OFN) intervention, *Coverage and Cost-of-Care Links* (*CC Links*), among patients with hematologic cancer and their caregivers who are at increased risk of experiencing financial toxicity (FT).

**METHODS:**

All patients who presented to the Division of Hematology and Bone and Marrow Transplant (BMT) at an National Cancer Institute–designated cancer center between April 2021 and January 2022 were screened for FT during inpatient and outpatient visits. Patients who screened positive for FT and met the inclusion criteria were recruited to participate in *CC Links* that provided financial navigation and assistance via a financial navigator. Caregivers of patients undergoing BMTs were also recruited to participate. Primary outcomes were defined as improvements in FT, distress, and physical and mental quality of life.

**RESULTS:**

Fifty-four patients and 32 caregivers completed the intervention and pre-/postintervention surveys. *CC Links* resulted in statistically significant decreases in the Comprehensive Score for FT for both patients (|*t*| = 2.42, *P* = .019) and caregivers (|*t*| = 2.43, *P* = .021) and total FT (|*t*| = 2.13, *P* = .041) and material conditions scores (|*t*| = 2.25, *P* = .031) for caregivers only. Only 27% of eligible patients participated in the study, whereas 100% of eligible caregivers participated. The majority of participants rated the intervention highly for acceptability (89%) and appropriateness (88%). An average of $2,500 (USD) in financial benefits was secured per participant via *CC Links.*

**CONCLUSION:**

*CC Links* was effective in decreasing FT among patients with hematologic cancer and their caregivers while demonstrating high acceptability and appropriateness ratings.

## INTRODUCTION

Rising costs of cancer care place patients with cancer and their caregivers at increased risk of experiencing financial toxicity (FT),^1^ a term referring to the psychological, material (eg, not being able to pay medical bills or going into debt), and behavioral (eg, delaying or forgoing medical care because of costs) aspects of financial hardship from cancer.^2^ FT is related to reduced quality of life (QOL), psychological distress, inadequate health care and treatment nonadherence, and potentially worse survival.^3-5^ Patients with hematologic cancer and their caregivers are especially vulnerable to FT because of hematologic cancer treatments, which often entail bone marrow transplantations (BMTs),^6,7^ lengthy hospital stays, prolonged intensive follow-up,^8,9^ and potential complications such as graft versus host disease.^10^ To meet treatment demands, patients and their caregivers often relocate to transplantation centers for a minimum of 3 months after a BMT, leading to periods of job and income loss for patients and caregivers.^11-13^

Oncology financial navigation (OFN) can address FT by helping patients optimize health insurance, identify assistance for out-of-pocket expenses, or apply for disability or family medical leave.^14,15^ Limited available research on OFN programs has reported positive impacts on patient and caregiver financial burden and anxiety related to costs of care^16^ and debt relief or financial benefits for patients .^17^ However, no OFN programs have been developed to meet the unique financial challenges experienced by both patients with hematologic cancer and their caregivers or tested in outpatient and inpatient settings (ie, actively undergoing BMT) where patients and caregivers may be most at risk for incurring FT.^18^ The goal of the current study was to test the feasibility, acceptability, and preliminary effectiveness of an OFN intervention for hematologic cancer survivors and their caregivers, *Coverage and Cost-of-Care* Links (*CC Links*).

## METHODS

### Study Design

This was a single-arm feasibility and acceptability trial conducted in the Division of Hematology and BMT at an National Cancer Institute–designated Cancer Center (ClinicalTrials.gov identifier: NCT05465577) at the University of Kentucky. Survey and electronic health record data were collected. Participants provided written informed consent. The study was approved by the University of Kentucky's Institutional Review Board (61653).

### *CC Links* Intervention

The intervention was developed using an iterative process^19^ and consisted of a financial navigator embedded in the Division of Hematology and BMT. The financial navigator interacted with patients and their caregivers (when applicable) in person and via telephone and electronic mail, as needed, to help address financial needs. The navigator's functions included the following: screening for FT to identify unmet financial needs (using the *FACIT-Comprehensive Score for Financial Toxicity* [*COST*])^20^ and the National Comprehensive Cancer Network's (NCCN) Distress Thermometer (DT) and Problem List,^21^ initiating cost-of-care conversations, providing cost-of-care estimates, assessing adequacy of health insurance coverage and assisting with applying for additional coverage, assisting with internal financial assistance program applications, connecting patients/caregivers with disease-specific resources and other external assistance programs, coordinating financial assistance services during discharge planning and during transitions between outpatient and inpatient settings, and referring patients to social workers and other staff/resources as needed.

### Sample

All patients who had a hematology and/or BMT appointment and were age 18 years or older were screened for FT by nurses and/or medical assistants on every inpatient hospital admission or outpatient clinic visit between April 2021 and January 2022. During this time frame, patients were screened for FT using paper versions of the COST and DT. Those who screened positive for FT (indicated by *COST* ≤ 24^22^ and/or scores >4 on the DT with selection of financial or insurance issues on the problem list^23^) and were able to read/write in English were eligible for the CC Links intervention. Patients who did not have a hematologic cancer diagnosis and/or were undergoing chimeric antigen receptor *T*-*cell therapy* were excluded from this study. The financial navigator initially contacted eligible patients via phone to share information about the study and schedule a meeting time to complete consents and surveys. In addition, the navigator also recruited eligible patients during hospital/clinic visits when possible if we were unable to make contact via phone. Only family or friends who self-identified as the caregiver of a patient undergoing BMT were eligible to participate in the *CC Links* intervention with the patient. This was done to help with recruitment and retention of caregivers since BMT patients are required to have a caregiver to assist with treatment and recovery. Respective caregivers were consented in person by the financial navigator during the BMT patients' hospital/clinic visit.

### Data Collection

Surveys were administered on paper and were collected in person by the financial navigator or via US mail or electronic mail. Participants completed a baseline survey during a hospital/clinic visit before participating in the *CC Links* intervention. Postsurveys were completed by participants after financial needs were addressed by the financial navigator or at the end of the study period (whichever came first).

#### Demographic and clinical data.

Patients and caregivers self-reported demographic data. Several variables were dichotomized for analysis, including health insurance status, education (high school or less *v* more education), employment (work outside the home *v* other work status), and time since cancer diagnosis (≥1 year *v* <1 year). Clinical data (eg, cancer diagnosis, time since diagnosis, treatments, and medications) were abstracted from the electronic health record.

#### Patient- and caregiver-reported outcomes.

Patient-reported FT related to cancer care was measured in three domains^24^: psychological response, material conditions, and coping behaviors using items from the Medical Expenditure Panel Survey-Experiences with Cancer Survivorship Supplement (MEPS-ECSS)^25^ and the *COST*.^20^

##### Psychological response.

The 11-item *COST* (α = .92)^20^ measures emotional aspects of FT. Each item is scored on a 5-point ordinal scale (0 = not at all to 4 = very much). Negative items were reverse-coded before creating the total *COST* score, with lower values indicating greater FT.

##### Material conditions.

Eight items (six from MEPS-ECSS^25^ and two from the demographic survey) were used to measure direct costs of cancer care (eg, borrowing money, going into debt, filing for bankruptcy, and unable to pay medical bills). Six yes/no items and two ordinal items were summed to create a total score, with higher scores indicating greater FT.

##### Coping behaviors.

A series of 16 yes/no MEPS-ECSS^25^ items related to foregoing care because of financial circumstances were used. A total score was derived from the sum of yes responses, with higher scores indicating greater use of coping behaviors. Item selection and scoring were based on a previous FT study; however, additional items from the MEPS-ECCS were used in the current study (see Appendix Table A1 [online only]).^26^

A *total FT* score was created on the basis of the three domains of FT^24^ using a reverse-coded total score for the *COST* (ie, reverse-coded from the original scoring so that higher scores are indicative of greater FT), the material conditions score, and the coping behavior score. Observed total scores were divided by the maximum possible total to create a percentile score for each instrument and were combined for each participant (range from 0 to 3), with higher scores indicating greater FT.

*Distress* was measured using the NCCN's DT.^21^ A cutoff score of four indicated clinically elevated distress levels.^23^ We also assessed whether financial/insurance problems were chosen from the DT problem list.

*Health-related QOL* was measured using four *Patient-Reported Outcomes Measurement Information System* (*PROMIS*) scales: *PROMIS physical health* and *mental health* subscales (from the 10-item *PROMIS* Scale v1.2—Global Health),^27^ the 4-item *PROMIS Anxiety* Short Form,^28^ and the 6-item *PROMIS Depression* Short Form.^29^ Higher scores indicate more of the construct being measured. Scoring was performed via *HealthMeasures* scoring service, and standardized values of the total scores (ie, t-scores) were used for analysis.

#### Intervention feasibility and acceptability.

Feasibility was defined as ≥ 60% enrollment of eligible participants (on the basis of existing OFN studies).^16,17^ Acceptability and appropriateness of the intervention were measured using the *Acceptability of Intervention* and *Intervention Appropriateness* measures.^30^ Each is composed of four items responded on a Likert-type scale (0 = completely disagree to 4 = completely agree; higher scores indicate greater acceptability and appropriateness). An additional six items from the *Treatment Acceptability and Preferences*^31^ survey further measured acceptability (eg, appropriateness, convenience, helpfulness, relevance, etc; 0 = not at all to 10 = extremely, with higher scores indicating greater acceptability).

### Data Analysis

Study data were summarized using means and standard deviations or frequency distributions. We calculated pre-/postintervention changes (pre minus post) in outcomes for patient and caregiver samples using paired t-tests. An a priori power analysis estimated that we would have at least 80% power to detect a medium effect size (*d* = 0.5), given at least 30 participants per group.^32^ For all measures except the *COST* and *PROMIS* health-related QOL measures, decreases pre- to postintervention indicate improvements in outcomes measured. We summarized the evaluation findings for each of the patient and caregiver groups separately; we also assessed this for the combined group to estimate overall participant perception. Data analysis was conducted using SAS, v. 9.4; an α level of 0.05 was used for inferential testing.

## RESULTS

**FIG 1.**
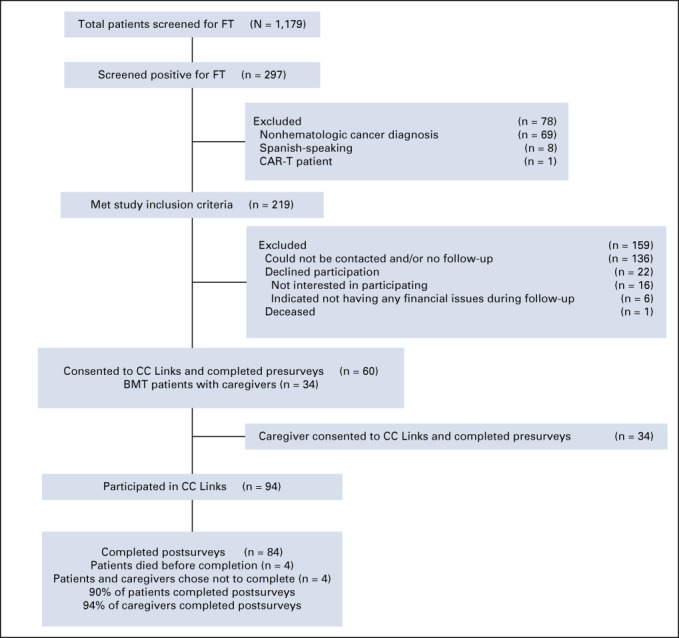
Patient and caregiver participation in CC Links. BMT, bone and marrow transplant; CC links, coverage and cost-of-care links; *COST*, comprehensive score for financial toxicity; DT, distress thermometer; FT, financial hardship.

A total of 1,179 patients with hematologic cancer were screened for FT (Fig 1). Two hundred and ninety-seven patients screened positive for FT, but only 219 were eligible for the study and were invited to participate in *CC Links.* Sixty patients (27%) consented to participation and completed presurveys. Roughly 57% of the patients who participated were recruited from the inpatient setting. Thirty-four caregivers of patients undergoing BMTs (100%) also consented to participate and complete preintervention surveys. Six patients passed away during the study (four before completing postintervention surveys). Two patients and two caregivers were lost to follow-up before completion of the postintervention survey. Fifty-four patients and 32 caregivers participated in the intervention and completed pre-/postsurveys.

### Demographic and Clinical Data

Most patients and caregivers identified as females with an average age of 63 years (Table 1). Nearly half of the patients had a high school education or less (45%), whereas only about one third of caregivers had this level of education (32%). Less than one fourth of the patients were employed (23%), whereas more than half of caregivers were employed (59%). Nearly all participants were insured, and roughly 32% were below the federal poverty level.

**TABLE 1.**
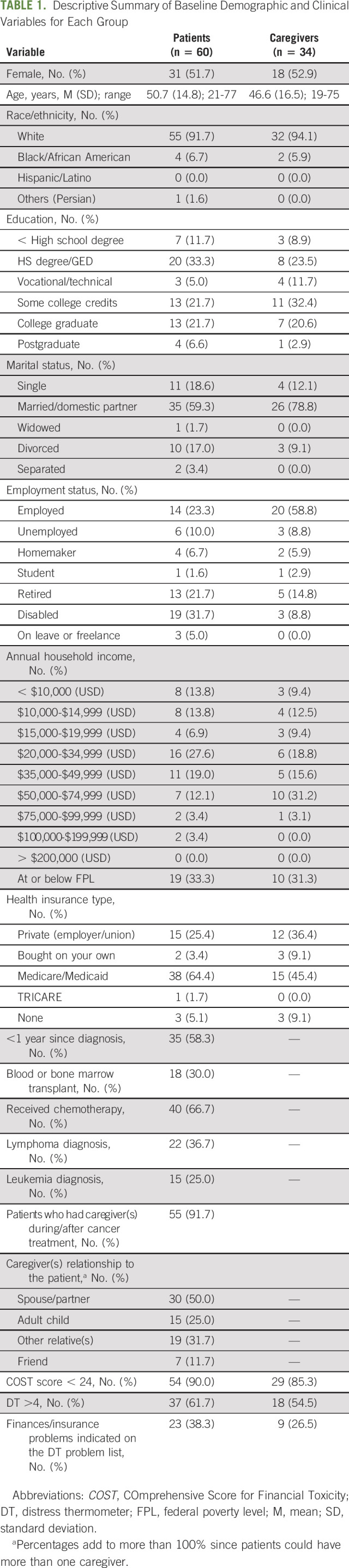
Descriptive Summary of Baseline Demographic and Clinical Variables for Each Group

More than half of patients were within their first-year postdiagnosis (58%), with lymphoma (37%) and leukemia (25%) being the most common cancers. Almost one third had received a BMT (30%), and two thirds had received some type of chemotherapy treatment (67%). The majority of patients (90%) and caregivers (85%) had a *COST* at or below 24 at baseline. Sixty-two percent of patients and 55% of caregivers had a DT score of > 4. Nearly 40% of patients and more than 25% of caregivers indicated problems with finances or insurance in the last week.

### *CC Links* Delivery and Processes

Study participants had an average of three in-person meetings (range 0-21) and five telephone interactions (range 1-23) with the financial navigator. The most common financial concerns were related to high out-of-pocket costs (ie, inability to afford medical bills). The most frequently provided OFN services were assisting with financial assistance programs and grants applications. The financial navigator was able to secure $124,600 (USD) in financial benefits for 48 participants via travel ($24,000 USD), urgent need ($16,000 USD), patient financial assistance ($9,100 USD), and co-pay assistance grants ($75,500 USD).

### Patient- and Caregiver-Reported Outcomes

Among patients, the only significant change from pre- to postintervention was in the psychological response score or COST (Table 2) with an average decrease of 2.30 points (*P* = .019; Hedges' *g* = 0.33). Among caregivers, there was a significant improvement in the *COST* (average decrease of 2.97 points, *P* = .021, *g* = 0.43), *material condition* scores (average decrease of 0.63 points; *P* = .031, *g* = 0.39), and *total FT* scores (average decrease of 0.13 points, *P* = .041, *g* = 0.37). There were no significant differences between patients and caregivers on any of the measures except for the PROMIS physical and mental health scores where caregivers scored higher than patients (|t| = 4.1, *P* < .001 and |t| = 2.2, *P* = .029 respectively; Table 3).

**TABLE 2.**
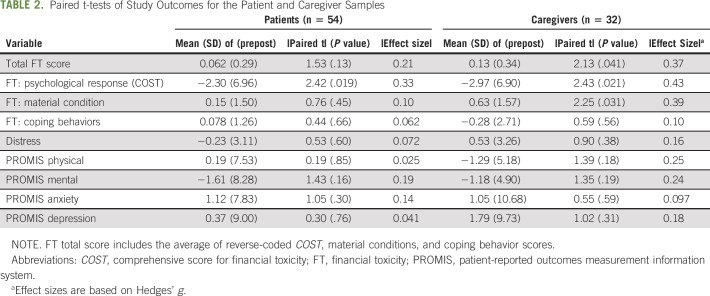
Paired t-tests of Study Outcomes for the Patient and Caregiver Samples

**TABLE 3.**
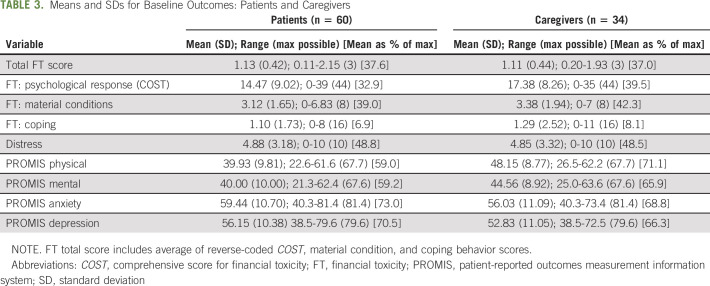
Means and SDs for Baseline Outcomes: Patients and Caregivers

### Feasibility, Acceptability, and Appropriateness

Enrollment was at ≥ 60% for caregivers (100%), but only 27% for patients. There was little variability between patients and caregivers in average ratings for *CC Links* acceptability (14.2, SD = 3.0; range 0-16 and 14.3, SD = 2.9; range 4-16, respectively), nor was there a difference in average ratings for appropriateness (14.2, SD = 2.9; range: 0-16 and 14.2, SD = 3.2; range: 4-16 respectively; Table 4). The average scores as a percentage of the maximum possible score of 16 were 89% and 88% for acceptability and appropriateness, respectively. The average total score on the six acceptability attributes (convenience, helpfulness, relevance, worthwhileness, likelihood of future use, and likelihood to recommend) was 53.3 (SD = 12.7; range: 0 to 60) for the combined sample. This mean value represents 89% of the maximum score of 60.

**TABLE 4.**
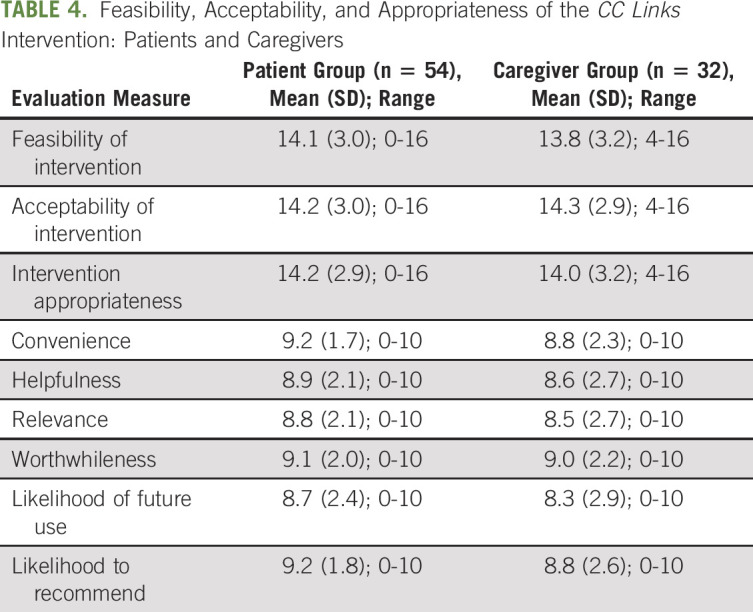
Feasibility, Acceptability, and Appropriateness of the *CC Links* Intervention: Patients and Caregivers

## DISCUSSION

To our knowledge, this study is the first to evaluate the feasibility, acceptability, and preliminary effectiveness of an OFN intervention, *CC Links*, designed specifically to address FT among patients with hematologic cancer and caregivers. We found high levels of baseline FT, distress, anxiety, and depression among those who enrolled in the intervention. The financial navigator's services helped secure a total of $124 (USD), 600 in financial benefits (roughly $2,500 (USD) per participant). Only 27% of eligible patients participated in the study, failing to meet feasibility thresholds, but 100% of eligible caregivers participated. High acceptability, appropriateness, and retention rates suggest that *CC Links* is acceptable to patients and their caregivers. Findings suggest that *CC Links* was effective in decreasing FT among patients with hematologic cancer and their caregivers.

High rates of FT coupled with the lack of well-defined care pathways to address FT care coordination during hematologic cancer treatment, especially in inpatient settings, were the impetus for our *CC Links* intervention. Patients with hematologic cancer experience some of the highest rates of FT of all patients with cancer, and their caregivers are similarly at elevated risk.^7-13,18^ 25 percent of the 1,179 patients screened in this study screened positive for FT. Despite high need for intervention, only 22% of those who screened positive and were approached were enrolled in the current study. Similarly, in a systematic review of 55 studies reporting FT among patients with hematologic cancer, 20%-50% experienced FT related to loss of work/income and savings and costs related to food and transportation.^18^ In an OFN intervention study by Knight et al,^33^ 243 of 683 patients with hematologic cancer who screened positive for FT were scheduled for receiving the intervention; however, only 107 (44%) participated in the intervention. Primary reasons for lack of participation in the current study were related to barriers in making contact with patients. Engagement was higher in the inpatient settings, which could be related to increased accessibility and minimized burden when interventions are provided alongside clinical care.^16^ Recruitment and intervention delivery timing were also key to engagement as those who had recently received a medical bill were more willing to participate.

*CC Links* was designed to meet the unique challenges of hematologic cancer treatment by establishing periodic and longitudinal FT screening for patients and addressing caregivers' financial hardship needs. Participating patients and caregivers rated the intervention highly in terms of acceptability and appropriateness. Intervention process data suggest that financial navigators for this participant population should be prepared to provide assistance with housing, transportation, and financial needs as well as navigating insurance plans and medical bills. Consistent with prior research, the most common OFN needs for both patients and caregivers were related to managing high costs of cancer care.^18^ In 2014, average cumulative costs for hematologic cancers paid by payers and patients combined ranged from $200,000 (USD) for chronic leukemias to more than $800,000 (USD) for acute leukemias in the first 36 months of treatment (compared with $250,000 (USD) for lung and $150,000 (USD) for colorectal cancers).^34^ As suggested in prior research, findings from the current study also indicate that caregivers may be more affected by the day-to-day financial implications of the patient's treatment and other caregiver-related out-of-pocket expenses for accommodations, transportation, and food and lost wages from work.^11,35^

Pre-/postintervention improvements in psychological response, material conditions, and total FT scores suggest that *CC Links* is a promising approach to addressing FT among patients and caregivers in hematologic cancer treatment settings. These findings address significant gaps in evidence on OFN: only two OFN interventions that included patients with hematologic cancer have been published.^32,36^ Furthermore, none has been designed for implementation with caregivers who are vulnerable to experiencing FT especially in inpatient settings.^18^ Outcomes for OFN interventions in hematologic and solid tumors are mixed, with only a few showing improvements in FT.^36,37^

The limited sample size, exclusion of non–English-speaking participants, and lack of random assignment limit generalizability of findings to other patients with cancer and caregivers. We were unable to evaluate difference in characteristics between eligible patients who did and did not participate, which would have provided insight into limited feasibility related to participant enrollment.

To our knowledge, our study is among the first to demonstrate the preliminary effectiveness of OFN among patients with hematologic cancer and their caregivers. Although we were unable to demonstrate feasibility, we were able to screen a large number of patients for FT using validated instruments and had high pre-/postintervention participation and survey response rates and high evaluation scores. To address feasibility limitations, future studies will benefit from targeted recruitment methods including standardized FT screening (ie, timing, personnel, and follow-up) and incorporating screening within electronic health records, which will also help with care coordination to address needs over time. Close collaboration and coordination with existing services and workflows are essential for the seamless integration of OFN interventions within health systems and help facilitate contact and communication with participants. Examples include partnering with psychosocial services to help maintain continuity of care when patients transition between inpatient and outpatient settings or with financial/billing departments to provide follow-up phone calls after medical bills are sent to patients. These strategies can also help address low engagement in outpatient settings where patients might have sporadic clinic visits. Future efficacy trials can benefit from using a factorial RCT design with multiple sites to help address challenges in randomly assigning for OFN services and provide a more rigorous evaluation of the effect of the intervention. In addition, multilevel interventions that address patient-, caregiver-, health system–, and policy-level factors are needed to advance the science of OFN and establish sustainable and effective ways to address FT of cancer.

## Figures and Tables

**TABLE A1.**
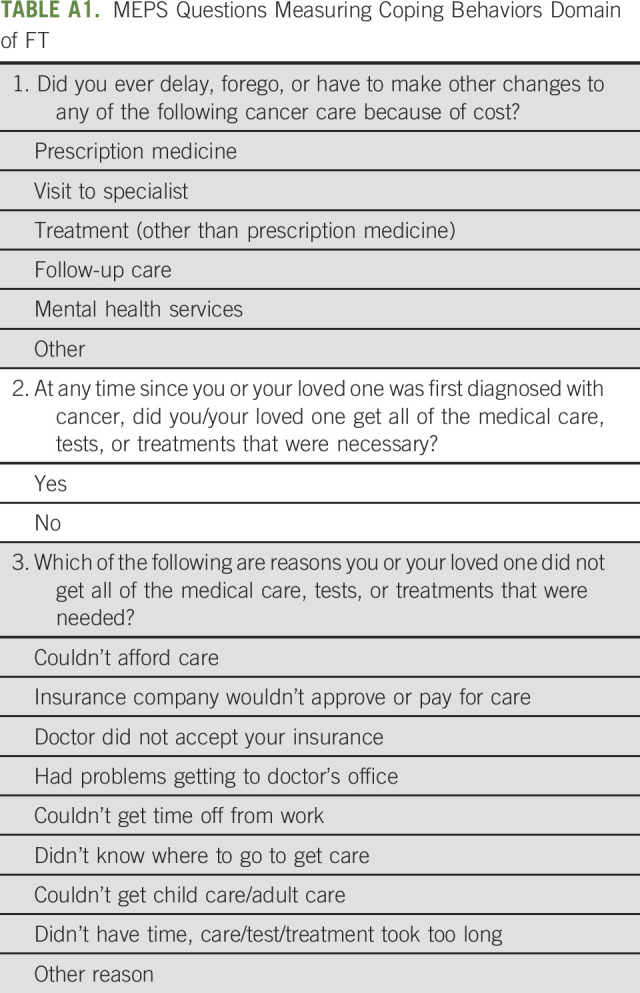
MEPS Questions Measuring Coping Behaviors Domain of FT
